# Is ambient heat exposure levels associated with miscarriage or stillbirths in hot regions? A cross-sectional study using survey data from the Ghana Maternal Health Survey 2007

**DOI:** 10.1007/s00484-017-1402-5

**Published:** 2017-07-26

**Authors:** Benedict Asamoah, Tord Kjellstrom, Per-Olof Östergren

**Affiliations:** 1 Social Medicine and Global Health, Department of Clinical Sciences, Lund University, Malmo, Sweden; 2 Centre for Technological Research and Innovation (CETRI), Limmasol, Cyprus; 3 Australian National University, Canberra, Australia

## Abstract

It is well established that high ambient heat could cause congenital abnormalities resulting in miscarriage or stillbirth among certain species of mammals. However, this has not been systematically studied in real field settings among humans, despite the potential value of such knowledge for estimating the impact of global warming on the human species. This study sought to test the hypothesis that maternal heat exposure during pregnancy in hot regions is associated with increased prevalence of spontaneous abortions or stillbirths and to develop an analytical strategy to use existing data from maternal health surveys and existing data on historical heat levels at a geographic grid cell level. A subsample of the Ghana Maternal Health Survey 2007 was used in this study. This study sample consisted of 1136 women with pregnancy experiences between 2004 and 2007, out of which 141 women had a pregnancy that terminated in miscarriage or stillbirth. Induced-abortion cases were excluded. The linkage between ambient heat exposure and pregnancy outcome followed the epidemiological time-place-person principle, by linking timing of pregnancy outcome with historical data of local area heat levels for each month, as estimated in an international database. Maternal heat exposure level was estimated using calculated levels of the wet-bulb globe temperature (WBGT), which takes into account temperature, humidity, heat radiation, and air movement over the skin (wind speed). The values we used applied to exposure in the shade or in buildings without cooling (no solar heat radiation) and a standard air movement of 1 m/s. We applied two exposure durations: yearly average and monthly average for second month of pregnancy. In one analysis, we restricted the sample to four regions with time-homogeneous ambient heat. Analysis was made using logistic regression. About 12% of the latest pregnancies ended in either miscarriage (9.6%) or stillbirth (2.8%). The odds ratios indicated 12 to 15% increase (OR_crude_ 1.15, 95% CI 0.92–1.42, and OR_age adjusted_ 1.12, 95% CI 0.90–1.39) in the odds of having a stillbirth or miscarriage with each additional degree increase in WBGT, although this was just outside two-sided statistical significance. The WBGT range was quite narrow (most annual values in the range 24–26 °C, and most monthly values in the range 23–27 °C), which may have hidden any real impacts of high heat levels. The seemingly positive association observed disappeared after adjusting for gravidity. The analyses of the four selected regions indicated 27 to 42% increase in the odds of experiencing miscarriage or stillbirth with every degree increase in WBGT (crude OR 1.42 95% CI 1.00–2.03). This association remained after adjusting for maternal age pregnancy history, although no longer statistically significant (adjusted OR 1.27, 95% CI 0.89–1.81). Environmental heat exposures may be associated with adverse pregnancy outcomes, but this study was inconclusive, possibly because the heat exposure range was small. Historical records of routine observations in existing databases can be used for epidemiological studies on the health effects of heat, although data from properly and purposively designed studies might be more suitable for further studies.

## Introduction

Climate change has emerged as a major challenge in the twenty-first century, and global warming with accompanied ambient heat rise of about 0.5 to 1 °C threatens the survival of biological species including humans (McMichael and Woodruff 2006; Poursafa et al. 2015; Weihua et al. 2015). Previous studies have suggested major harmful effects of climate change and ambient heat changes on human health (Amegah et al. 2016; Mboera et al. 2011; Sheffield and Landrigan 2011) including transmission of vector-borne infectious diseases such as malaria, dengue, and leishmaniasis (Wu et al. 2016); and diseases and mortality related to heat stress (Bunker et al. 2016; Matte et al. 2016). Although the effects of climate change on human health are widely evident, studies on the impact of climate change and pregnancy outcomes are limited (Basu et al. 2010; Van Zutphen et al. 2012) and the magnitude of the risk and its effect on pregnancies are hardly recognized (Poursafa et al. 2015; Strand et al. 2011). Although the impact of maternal exposure to the external environment may not be a direct cause of adverse outcomes related to pregnancy, possible mechanisms of action through genetic, epigenetic, socio-demographic, and other known susceptibility factors cannot be ruled out.

The human body through homeostasis maintains its body temperature at around 37 °C (±1 °C) (Ziskin and Morrissey 2011). Humans can tolerate internal temperatures below 35 or above 41 °C for only very brief periods of time. The largest source of heat to the body results from metabolic heat production. However, heat can also be gained from the environment through radiation, convection, or conduction when the external temperature exceeds the skin temperature and vice versa, when the skin-to-air thermal gradient is reversed. Thus, when a person is exposed to warm environmental conditions and the body temperature exceeds a theoretical “set-point,” the body offsets its thermostatic control to stabilize its internal temperature through cooling mechanisms such as increased blood flow to the skin and sweating. When the temperature falls below the set-point, the reverse reaction occurs, i.e., heat gain processes such as decreased blood flow to the skin and shivering set in. Thus, under normal physiological conditions, the set-point is relatively stable and unaffected by work or environmental temperature. There exist differences between individuals and ethnic groups in the way they acclimatize to heat, which is mainly based on differences in certain characteristics which might influence heat transfer such as surface area, height/weight ratio, thickness of insulating skin fat layers, and in physical ability to produce work and heat. High ambient heat may cause physiological disorders such as heat syncope, heat edema, heat cramps, heat exhaustion, and heat stroke (the latter three are of clinical importance). The mechanisms underlying these systemic disorders are circulatory insufficiency, water and electrolyte imbalance, and/or hyperthermia (high body temperature). There could also be local disorders such as skin lesions. Populations known to be at increased risk of developing heat disorders include infants, elderly (especially the poor and those with chronic conditions), and healthy individuals who attempt prolonged physical exertion or a exposed to excessive heat stress (International Labour Office 1998).

The effect of pregnancy on the women’s heat tolerance is not clear, but altered hormone levels and the increased circulatory demands of the fetus on the mother may increase her susceptibility to heat disorders. “Severe maternal hyperthermia (over-heating) due to illness appears to increase the incidence of fetal malformation, but there is no evidence of a similar effect from occupational heat stress” (International Labour Office 1998). However, the human embryo and fetus can be susceptible to elevated temperatures during certain stages of development such as cell proliferation, cell migration, cell differentiation, and apoptosis (programmed cell death) at different times for different organ structures. The embryo and developing fetus have limited capacity to regulate its own temperature and depend entirely on the mother’s thermoregulatory capacity. Maternal elevated temperatures above the normal set-point have been found to trigger developmental abnormalities in animal models. However, due to the different thermoregulatory mechanisms for humans and the thermoneutral ambient temperatures, these effects and the thresholds cannot be directly extrapolated to humans. Nonetheless, previous studies have suggested that smaller levels of thermal stress in the mother, although may be asymptomatic, could theoretically result in increased redirection of blood to the periphery (as heat loss mechanism) at the expense of placental and umbilical blood perfusion, which could eventually reduce heat exchange with the fetus (Ziskin and Morrissey 2011).

A recent systematic review regarding adverse birth outcome of climate change, taking a broad range on environmental effects into account, suggests significant associations between the studied climate change variables and the pregnancy-related outcomes such as eclampsia, preeclampsia, congenital cataract, low birth weight, preterm birth, sex ratio, and length of pregnancy (Poursafa et al. 2015). The abovementioned review analyzed studies that included either one or both of the two temperature spectrums: cold and hot. Whereas most studies associate eclampsia and preeclampsia with cold and humid temperatures, temperature increases have been related to preterm birth (Basu et al. 2010; Carolan-Olah and Frankowska 2014; Strand et al. 2012). It is estimated that about 10–15% of the congenital structural abnormalities are as a result of environmental factors on fetal development (Brent 2001; Gilbert-Barness 2010). An environmental factor can produce a permanent abnormality in structure or function, restriction of growth, or death of the embryo or fetus that could lead to miscarriage or fetal death resulting in stillbirth (Gilbert-Barness 2010). Fetal exposure to maternal hyperthermia (e.g., fever because of an infectious disease) at 7 to 16 weeks of gestation has been found to result in severe fetal embryonic malformations in humans. Several studies have concluded that the teratogenicity of maternal hyperthermia is most profound in the first trimester (Dreier et al. 2014; Fraser and Skelton 1978; Pei et al. 2015).

Dadvand et al. (2011) found that exposure to hot weather or thermal stress was associated with 5 days decrease in the average pregnancy duration (Dadvand et al. 2011). Previous studies have suggested that one of the factors related to preterm births is the secretion of oxytocin and heat-shock proteins that result from heat stress (Dreiling et al. 1991; Poursafa et al. 2015). Dehydration caused by thermal heat exposure has also been hypothesized as another possible reason (Shoghag et al. 1997).

In summary, hyperthermia is a well-known teratogen in animals and maternal hyperthermia has been associated with birth defects in human (Edwards 2006; Edwards et al. 2003; Moretti et al. 2005; Van Zutphen et al. 2012). The central nervous system is known to be most sensitive to elevated temperatures (Edwards 1998; Edwards et al. 2003; Graham 2005; Moretti et al. 2005). In human clinical studies, an oral temperature of 37 °C (98.6 °F) is considered normal, and 39 °C (102 °F) is regarded as the threshold for potential damage (Van Zutphen et al. 2012). However, the teratogenicity of heat is dependent on the overlap between the ambient heat elevation and the susceptible stage of development; the type of defect depends on the particular stage of development (Edwards 1998, 2006; Graham 2005; Van Zutphen et al. 2012). Major birth defects may result in miscarriage or fetal death (Parker et al. 2010), as it is known that a large proportion of embryos with neural tube defects expires in the uterus prior to expulsion or delivery (Edwards et al. 1995). Ambient heat in hot regions of the world could therefore induce congenital malformations in humans (especially for outdoor workers, e.g., in agricultural work), resulting in an increased prevalence of spontaneous abortion or stillbirth than would be expected from other causes acting alone, but few empirical investigations in real-world settings are reported in the existing scientific literature. The aim of this study was to use an existing maternal health database to test the hypothesis that maternal heat exposure during pregnancy in hot regions is associated with increased prevalence of spontaneous abortions or stillbirths, and to develop an analytical strategy to use existing data from maternal health surveys and existing data on historical heat levels at a global grid cell level (Kjellstrom et al. 2017).

## Method

### Study design and data collection

This study used miscarriage and stillbirth reported in the Ghana Maternal Health Survey (GMHS), 2007, as an outcome for assessing the association between elevated ambient heat and adverse birth outcomes. The GMHS 2007 is the first nationally representative survey to collect comprehensive information on maternal morbidity and mortality in the country. The survey covered 1600 clusters (approximately 150 households per cluster) selected from the 10 administrative regions of Ghana across urban and rural areas. The survey was conducted in two phases. Phase 1 collected general information on households with maternal deaths, whereas phase 2 followed with a verbal autopsy into the causes of maternal deaths. A separate women’s questionnaire was administered to women 15–49 years in a subsample of households to collect information on maternal health-related issues including pregnancies, live births, abortions and miscarriages, and utilization of health services in relation to these events. This study used data from responses to the women’s questionnaire. Details of the study design and maternal data collection process have been published in a technical report (Ghana Statistical Service (GSS) Ghana Health Service (GHS), and Macro International 2009).This study sample consisted of 1136 women with pregnancy experiences between 2004 and 2007, out of which 141 cases had a pregnancy that terminated in miscarriage or stillbirth. Induced-abortion cases were excluded.

## Variables

### Dependent variable

#### Pregnancy outcome

Pregnancy outcome was limited to the last pregnancy in the 3 years preceding the survey (2004–2007). There were four response alternatives namely “born alive,” “born dead,” “miscarriage,” and “induced-abortion.” The response alternatives were dichotomized as either “live birth” or “miscarriage/stillbirth.” Induced-abortions outcomes were excluded from this analysis. To determine time and place for exposure, we used information in the survey regarding the women’s domicile and reported time for the adverse pregnancy outcome.

### Maternal exposure to heat

To establish the heat exposure level in this study, we used the environmental wet-bulb globe temperature (WBGT). WBGT is a heat index based on temperature, humidity, wind speed, and solar radiation data that has been collected over a long period of time. The WBGT heat stress is calculated by the Liljegren method where climatic variables of temperature, humidity, solar radiation, and wind speed are used in a rational thermodynamic heat exchange model, with approximations for no direct sunlight, and wind speed of 1 m/s (Liljegren et al. 2008). The formulas are based on indoor conditions or outdoors in the shade. The WBGT values were obtained from the “high occupational temperature health and productivity suppression” (HOTHAPS) program database [31] and including WBGT heat levels calculated from the Climate Research Unit (CRU at University of East Anglia, UK) global grid cell-based climate variable data. The HOTHAPS database website ClimateCHIP.org (ClimateSoft Ltd. n.d.) and the associated HOTHAPS-Soft database contain several key climate variables (temperatures, dew point, wind speed, and rain) as well as two widely used heat stress indexes WBGT and UTCI (Universal Thermal Climate Index), using publicly available grid cell and weather station recordings (NOAA’s *Global Summary of the Day*, GSOD, and CRU 3.1 gridded monthly averages for comparison and data quality assurance) over a time period from 1980 to the present. Maternal exposure to heat was estimated using the grid cell based WBGT values available on the climate chip website. WBGT is important for the body’s ability to lower the temperature by sweating. The linkage between ambient heat and pregnancy outcome followed the epidemiological time-place-person principle, by linking time of pregnancy outcome with the grid cells from the HOTHAPS database, which contains historical heat exposure level (WBGT) data. Thus, the WBGT was linked to place and time of the event, namely, where and when the pregnancy ended. However, in this study, determination of the women’s domicile could only be made at the level of administrative region of Ghana in the available maternal health survey dataset. The 10 regions in Ghana geographically overlap the grid cells (span over different heat exposure levels), which blunted the estimation of mean WBGT heat exposures on the women. For this reason, we restricted the final analysis to the four regions, two in the north and two in the south, which were reasonably homogenous and represented a clear contrast regarding mean WBGT values. Thus, we tried in two steps to narrow down the exposure window as well as to increase the exposure contrast by restricting the sample to the four regions with time-homogeneous heat exposure levels despite the potential power problems due to low number of pregnancy events in the selected regions. The exposure points were determined by estimating the month and year that the pregnancy termination occurred, the duration of the pregnancy, and using that to determine the WBGT exposure window at the second month of pregnancy in the region of residence. The exposure window was narrowed to the first months of pregnancy based on well-known evidence that the teratogenicity due to maternal elevated temperatures occur between months 1 and 3 of gestation (Dreier et al. 2014; Fraser and Skelton 1978; Pei et al. 2015; Shiota and Opitz 1982). Aside the WBGT values at the second month of pregnancy, we also determined the yearly WBGT distribution based on the place and year of pregnancy. This was to test whether it mattered if we tried to specify the exposure window in time or not. We mainly used the yearly average WBGT (WBGT mean) values for our analyses and repeated similar analyses using the yearly maximum WBGT (WBGT max) values.

### Maternal characteristics

#### Maternal age at termination of pregnancy

This variable was computed from the year of birth and year that the pregnancy ended.

#### Area of residence

Area of residence was classified as urban and rural.

#### Educational level

Highest level of education attained was categorized into four groups as “up to primary school,” “junior secondary school,” “senior secondary school,” and “higher education.”

### Pregnancy history

Pregnancy history variables included *Gravidity* (total number of pregnancies), *having ever had a miscarriage and number of miscarriages, having ever had a stillbirth,* and *number of stillbirths*.

### Use of maternal health services

#### Antenatal visits

The number of antenatal visits was classified as one, two to three, and four or more.

#### Reason for seeking antenatal care

Reason for seeking antenatal care was dichotomized as “because of a problem” and “just for checkup.”

### Data analysis

**Fig. 1:**
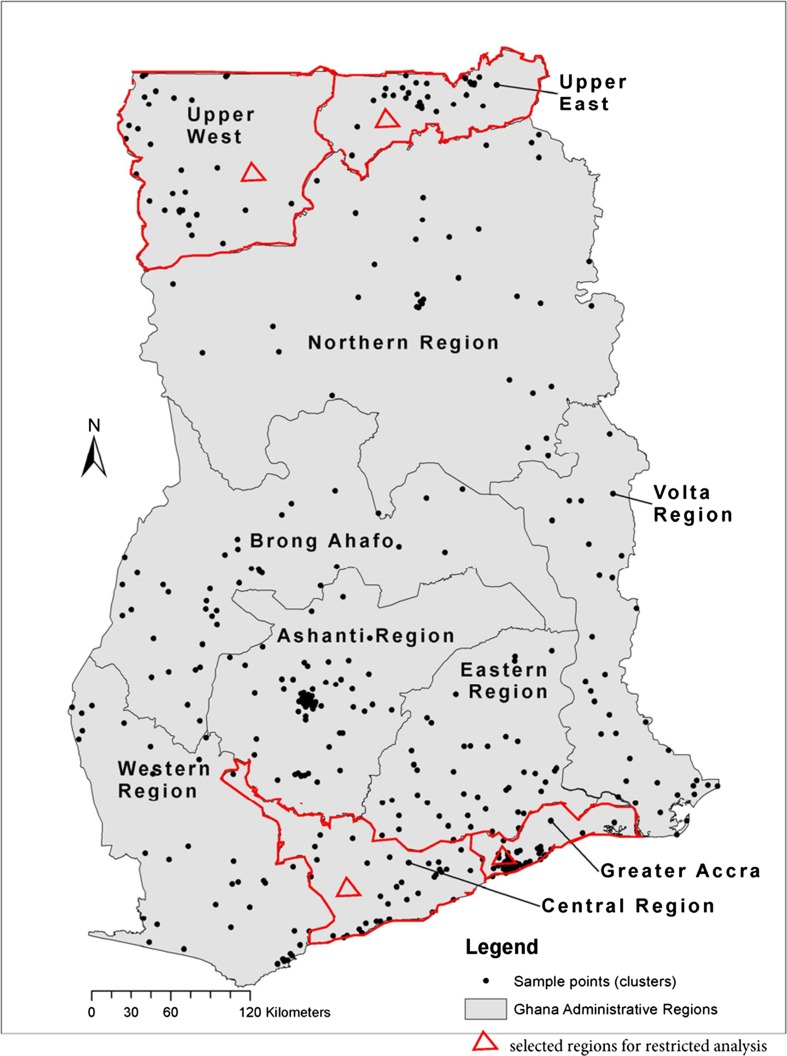
Map of Ghana showing the 10 administrative regions and 10 selected regions for restricted analysis

The climate data was incorporated into data from the Ghana Maternal Health Survey 2007 and analyzed using the software SPSS version 22. Frequency tables and cross-tabulations were used to describe the data. Simple and multiple (stepwise) logistic regression analyses were used examine the association between maternal heat exposure and pregnancy outcomes, adjusting for possible confounders. The analyses were first performed using respondents from all the 10 administrative regions of Ghana and then a restricted analyses was done using 4 selected regions based on geographical locations; two in the north (Upper East and Upper West regions) and two in the south (Greater Accra and Central regions). Figure 1 shows the map of Ghana indicating the 10 administrative regions and the 4 selected regions mentioned previously.

## Results

**Fig. 2:**
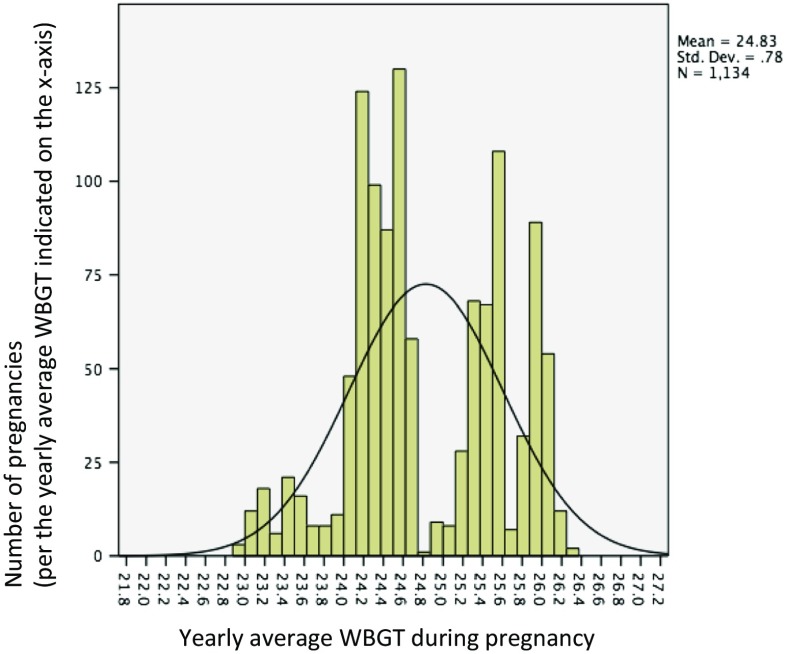
Yearly average ambient heat (Mean WBGT) during pregnancy among 1134 Ghanaian women with pregnancy history between 2004 and 2007 (based on the year pregnancy ended)

**Fig. 3:**
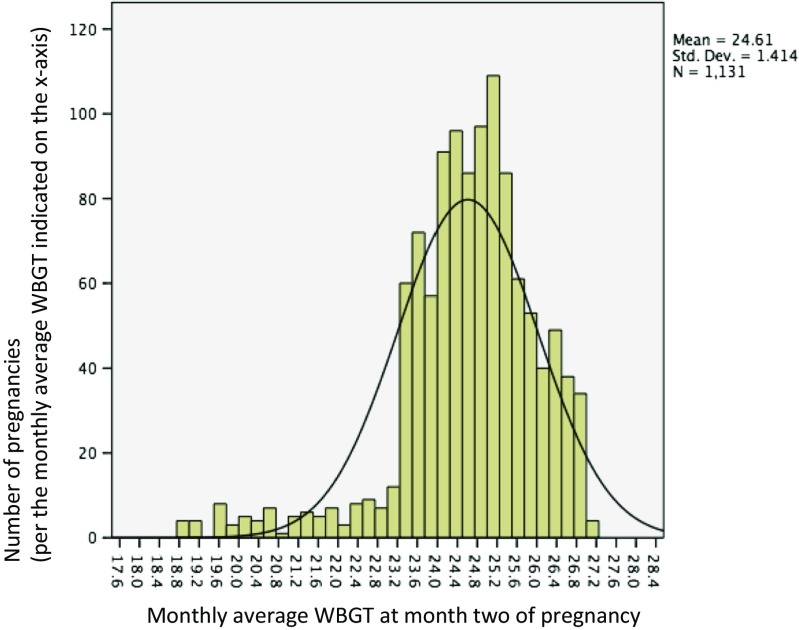
Monthly average ambient heat (Mean WBGT) during the second month of pregnancy or at the end of pregnancy (for pregnancies terminated before the second gestational month) among 1131 Ghanaian women with pregnancy history between 2004 and 2007

Table 1 gives a description of the sample characteristics. Of the 1136 respondents, majority were young women (mean age 22.9 years, median 22.0 years), lived in rural areas (54%), and had low level of education. About 12% of the latest pregnancies ended in either miscarriage (9.6%) or stillbirth (2.8%). Similarly, about 12% of the women had ever had a miscarriage, whereas a little more than 3% had ever had a pregnancy that ended up in stillbirth. The majority of the women in our study were primigravida. Figures 2 and 3 give the distribution of heat exposure levels (WBGT) at the end of the pregnancy in the study sample, based on yearly distribution and the second month of pregnancy, respectively. The yearly WBGT values show a bimodal distribution, which reflects a combination of place and time.

**Table 1:** Sample characteristics of 1136 Ghanaian women with pregnancy experiences between 2004 and 2007, drawn from the Ghana Maternal Health Survey 2007

Variable names	Categories	Frequency	Valid percent (%)
Age pregnancy ended	Mean, [median] (SD)	22.87, [22.00] (4.2)	
Area of residence	Urban	520	45.8
	Rural	616	54.2
Educational level	Junior secondary school	503	44.3
	Senior secondary school	109	9.6
	Higher education	49	4.3
Total number of pregnancies	1	806	71.0
	2	285	25.1
	3	36	3.2
	4	9	0.8
Ever had miscarriage	Yes	133	11.7
	No	1003	88.3
Number of miscarriages	1	110	82.7
	2	17	12.8
	3	6	4.5
Ever had a still birth	Yes	39	3.4
	No	1097	96.6
Number of stillbirths	1	34	87.2
	2	5	12.8
Pregnancy outcome	Born alive	995	87.6
	Born dead	32	2.8
	Miscarriage	109	9.6
Pregnancy outcome (dichotomized)	Live birth	995	87.6
	Stillbirth or Miscarriage	141	12.4
Antenatal visits	1	42	4.1
	2–3	164	15.8
	4+	829	80.1
Reason for seeking antenatal care	Because of a problem	168	16.2
	Just for checkup	867	83.8
Year pregnancy ended	2004	257	22.6
	2005	305	26.8
	2006	278	24.5
	2007	296	26.1
Gestational age at end of pregnancy (in months)	1	15	1.3
	2	27	2.4
	3	33	2.9
	4	13	1.1
	5	9	0.8
	6	12	1.1
	7	4	0.4
	8	5	0.4
	9	1018	89.6

Table 2 shows the distribution of pregnancy outcomes according to maternal background characteristics, pregnancy history, and use of antenatal care services. Among women who lived in rural areas, about 9% reported that their latest pregnancies resulted in stillbirths or miscarriage compared to 16% of the urban residents. The proportion of stillbirths or miscarriages increased gradually with increasing levels of education. Women with higher number of pregnancies generally had higher proportions of miscarriages or stillbirths. The proportion of miscarriages or stillbirths was similar across the study years but varied by the calendar month that pregnancy ended. Living in a rural area was found to be significantly associated with lower odds of experiencing miscarriage or stillbirth (crude OR 0.55, 95% CI 0.38–0.78).

**Table 2:** Distribution of pregnancy outcomes (live birth versus miscarriage/stillbirth) according to maternal background characteristics, pregnancy history, and use of antenatal care services among 1136 Ghana women drawn from the Ghana Maternal Health Survey 2007

Variable	Categories	Live birth *N* (%)	Miscarriage or stillbirth *N* (%)	Total (%)	Crude OR (miscarriage or stillbirth)	95% CI
Age pregnancy ended	Continuous				1.07*	1.03–1.12*
Area of residence						
	Urban	437 (84.0)	83 (16.0)	520 (100)	Ref	
	Rural	558 (90.6)	58 (9.4)	616 (100)	0.55*	0.38–0.78*
	Total	995 87.6)	141 (12.4)	1136 (100)		
Educational level						
	Primary	224 (88.9)	28 (11.1)	252 (100)	0.64	0.27–1.50
	Junior secondary	433 (86.1)	70 (13.9)	503 (100)	0.83	0.37–1.84
	Senior secondary	92 (84.1)	17 (15.6)	109 (100)	0.95	0.38–2.37
	Higher education	41 (83.7)	8 (16.3)	49 (100)	Ref	
	Total	790 (86.5)	123 (13.5)	913 (100)		
Total number of pregnancies					2.44*	1.89–3.16*
	1	740 (91.8)	66 (8.2)	806 (100)		
	2	229 (80.4)	56 (19.6)	285 (100)		
	3	18 (50.0)	18 (50.0)	36 (100)		
	4	8 (88.9)	1 (11.1)	9 (100)		
Antenatal visits						
	1	38 (90.5)	4 (9.5)	42 (100)	1.5	0.52–4.39
	2–3	157 (95.7)	7 (4.3)	164 (100)	0.64	0.29–1.43
	4+	775 (93.5)	54 (6.5)	829 (100)	Ref	
	Total	970 (93.7)	65 (6.3)	1035 (100)		
Year pregnancy ended						
	2004	226 (87.9)	31 (12.1)	257 (100)		
	2005	264 (86.6)	41 (13.4)	305 (100)		
	2006	246 (86.6)	32 (11.5)	278 (100)		
	2007	259 (87.5)	37 (12.5)	296 (100)		
Month pregnancy ended						
	January	74 (85.1)	13 (14.9)	87 (100)		
	February	60 (78.9)	16 (21.1)	76 (100)		
	March	99 (89.2)	12 (10.8)	111 (100)		
	April	89 (85.6)	15 (14.4)	104 (100)		
	May	95 (94.1)	6 (5.9)	101 (100)		
	June	92 (80.7)	22 (19.3)	114 (100)		
	July	91 (93.8)	6 (6.2)	97 (100)		
	August	79 (81.4)	18 (18.6)	97 (100)		
	September	75 (84.3)	14 (15.7)	89 (100)		
	October	95 (91.3)	9 (8.7)	104 (100)		
	November	82 (96.5)	3 (3.5)	85 (100)		
	December	63 (90.0)	7 (10.0)	70 (100)		
	Total	994 (87.6)	141 (12.4)	1135 (100)		
Gestational age at end of pregnancy (in months)						
	1	0	15 (100)	15 (100)		
	2	0	27 (100)	27 (100)		
	3	0	33 (100)	33 (100)		
	4	0	13 (100)	13 (100)		
	5	0	9 (100)	9 (100)		
	6	0	12 (100)	12 (100)		
	7	0	4 (100)	4 (100)		
	8	0	5 (100)	5 (100)		
	9	995 (97.7)	23 (2.3)	1018 (100)		
	Total	995 (87.6)	141 (12.4)	1136 (100)		

*OR* odds ratio, *CI* confidence interval

* statistically significant at ﻿*p*﻿ <.05

Tables 3 gives the crude and adjusted odds ratios, with 95% confidence intervals from the stepwise multiple logistic regression analyses for the entire sample. Using the yearly WBGT averages, the crude odds ratio indicated a 15% increase (OR 1.15, 95% CI 0.92–1.42) in the odds of having an adverse pregnancy outcome (stillbirth or miscarriage) with each additional degree increase in atmospheric heat exposure level, although this was not statistically significant. The odds ratio decreased progressively after adjusting for maternal age at end of pregnancy and total number of pregnancy history (gravidity). The odds of having a miscarriage or stillbirth increased by more than twice with each additional pregnancy in the fully adjusted model (Table 3, model 2, adjusted OR 2.46, 95% CI 1.89–3.20). One-year increase in maternal age at pregnancy was associated with 8% increase in the odds of having stillbirth or miscarriage (Table 3, model 2, adjusted OR 1.08, 95% CI 1.03–1.12). When we modeled using the WBGT based on more narrow exposure window, monthly distributions, the results did not reveal any differences in the outcome (crude OR 1.03, 95% CI 0.84–1.27).

**Table 3:** Simple and multiple (stepwise) logistic regression analyses of the association (OR, 95% CI) between exposure to high ambient heat and miscarriage/stillbirth, adjusted for maternal age at pregnancy, and total number of pregnancies among 1135 women drawn from all 10 administrative regions of Ghana

	Crude OR (95% CI)	Model 1 OR (95% CI)	Model 2 OR (95% CI)
Ambient heat (Mean WBGT) based on yearly distributions	1.15 (0.92–1.42)	1.12 (0.90–1.39)	1.00 (0.80–1.25)
Ambient heat (Mean WBGT) based on month 2 of pregnancy	1.03 (0.84–1.27)		
Maternal age at end of pregnancy		1.07 (1.03–1.12)*	1.08 (1.03–1.12)*
Total number of pregnancies			2.46 (1.89–3.20)*

Model 1: adjusted for maternal age at end of pregnancy. Model 2: adjusted for maternal age at end of pregnancy and total number of pregnancies

*OR* odds ratio, *CI* confidence interval

﻿* statistically significant at *p ﻿*<.05

Tables 4 uses the four selected administrative regions (two in the north, and two in the south) to show the association between atmospheric heat exposure level (WBGT) and adverse pregnancy outcomes (miscarriage or stillbirth). This was an attempt to have a more focused exposure window using the four regions with time-homogenous heat exposure levels. The crude odds ratio shows each degree increase in wet-bulb globe temperature is associated with 42 % increase in the odds of experiencing miscarriage or stillbirth (OR 1.42, 95% CI 1.00–2.03). The odds ratio decreases successively but gradually after adjusting for maternal background characteristics (OR 1.36, 95% CI 0.95–1.95; adjusted for age, OR 1.27, 95% CI; adjusted for age). Contrary to the analyses with the entire sample (10 administrative regions), the analyses of the four selected regions showed a positive association even after adjusting for pregnancy history, although not statistically significant when controlling for age and number of pregnancies. Similarly to Table 3, the odds of having a miscarriage or stillbirth increased by about two times (OR 2.09, 95% CI 1.30–3.34) with each additional pregnancy experience. In the same way, 1-year increase in maternal age at pregnancy is associated with 10 % adjusted (OR 1.10, 95% CI 1.02–1.18) increase in the odds of having miscarriage or stillbirth as was found in the entire sample.

**Table 4:** Simple and multiple (stepwise) logistic regression analyses of the association (OR, 95% CI) between exposure to high ambient heat and miscarriage/stillbirth, adjusted for maternal age at pregnancy, and total number of pregnancies among 393 women drawn from all four selected regions of Ghana

	Crude OR (95% CI)	Model 1 OR (95% CI)	Model 2 OR (95% CI)
Ambient heat (Mean WBGT) based on yearly distributions	1.42 (1.00–2.03)*	1.36 (0.95–1.95)	1.27 (0.89–1.81)
Ambient heat (Mean WBGT) based on month 2 of pregnancy	0.93 (0.77–1.12)		
Maternal age at end of pregnancy		1.08 (1.01–1.16)*	1.10 (1.02–1.18)*
Total number of pregnancies			2.09 (1.30–3.34)*

Model 1: adjusted for maternal age at end of pregnancy. Model 2: adjusted for maternal age at end of pregnancy and total number of pregnancies

*OR* odds ratio, *CI* confidence interval

* statistically significant at *p *<.05

Similar analyses as mentioned previously were also done using the yearly maximum WBGT values, and the results were not appreciably different from that described previously. From the analysis of the 10 administrative regions, the odds of having a miscarriage or stillbirth were 0.97 (crude OR 0.97, 95% CI 0.57–1.62). When adjusted for maternal age and number of pregnancies, the odds ratio decreased slightly to 0.82 (95% CI 0.48–1.40). The analyses of the four selected regions showed a crude OR of 2.17 (95% CI 0.69–6.82) and an adjusted OR of 1.90 (95% CI 0.62–5.85) [These results are not shown in the tables.].

Also, we performed a restricted analysis with only primigravida in an attempt to reduce the potential impact of gravidity but the pattern was very similar to the results reported above (These results are not shown.).

## Discussion

The findings of this study suggest possible association between atmospheric heat exposure and adverse pregnancy outcomes: miscarriage or stillbirth that could be further explored. The likelihood of experiencing a miscarriage or stillbirth increased twofolds with each additional pregnancy, although the effect was not statistically significant after adjustment for possible confounders. The findings are supported by findings from previous studies elsewhere (Strand et al. 2012, 2011).

Our study shows that it is possible to make this type of analyses using available data. However, potential limitations have to be considered and factored in when interpreting the findings, and when designing future studies to further explore the health effects of high ambient heat. One important limitation in this study was the difficulty in quantifying actual heat exposure and accurately linking that to pregnancy outcomes in the available datasets. Even though the maternal health survey provides a very good data on maternal health and pregnancy outcomes, geographical data (GPS data) was not collected as part of the survey. The lack of specific geographical data presented a challenge for linking the maternal health data to a specific geographical location. Thus, the mean WBGT we used were regional averages based on the women’s region of residence. We limited the year pregnancy ended to the last 3 years before the survey year on assumption that a shorter period will limit the effect of internal mobility on our results. However, although there is a limited likelihood, there still could be some women who might have changed region of residence at the time of the survey and this could have biased our findings to a smaller extent.

Although we suspect that most malformed fetuses would end up in miscarriage or stillbirth, it is not all miscarriages or stillbirths that are due to malformations. The reverse is also true that it is not all malformations that end up in miscarriage or stillbirth (Edwards et al.; Moretti et al. 2005). Thus, lack of comprehensive data on pregnancy outcomes, including fetal malformations, limited the analysis regarding adverse pregnancy outcomes in this study. Pregnancies that ended in induced-abortions were excluded, since they could not be directly linked to adverse pregnancy outcomes. However, there could be a subsample of women who might have undergone elective termination of the pregnancy upon detection of fetal malformation during pregnancy (Cragan and Khoury 2000).

The pattern for the crude odds ratios observed for rural/urban residence, education, antenatal care attendance, and miscarriage or stillbirth (Table 2) is suggestive that (1) selection bias due to high maternal mortality among women of low socio-economic status is highly probable, (2) these variables could be a proxy of exposure to high ambient heat, and (3) there could be high potential for differential diagnosis favoring high socio-economic status women. Therefore, to avoid the potential biases and over-adjustment in our models, rural-urban residence status, education, and number of antenatal care (ANC) visits were not included in the adjusted models. Detailed explanations in relation to the above are discussed as follows.

The finding from this study that women resident in rural areas have reduced likelihood of adverse pregnancy outcomes related to miscarriage or still birth could be due to the high maternal mortality in the rural areas of Ghana (Asamoah et al. 2011), such that women with adverse pregnancy outcomes in rural areas hardly survive compared to those from urban settings. Since the women’s questionnaire used in the Ghana Maternal Health Survey 2007 was administered to surviving women, there is a possibility of selection bias associated with maternal deaths from complications due to fetal abnormalities. It could also be that most of the people interviewed in the urban areas are the less privileged urban dwellers with worse situations. Another alternative explanation could also be that most of these high-risk pregnancies or births are better captured by healthcare facilities in urban settings that have access to proper prenatal diagnosis techniques.

The number of antenatal visits was not found to be significantly associated with pregnancy outcome contrary to what we expected. The number of antenatal visits ranged from 1 to 44 in the study sample. Thus, there are some women in the category “4+ visits” who visited ANC at unusually high rates (up to 44 times), which could be due to complications. Therefore, there could be some misclassification emanating from classifying ANC visits simply into three categories as: one , two to three, and four or more. We tried different classification options, but that did not impact on the observed results.

Women who experience miscarriage or stillbirths may be more likely to get pregnant again as a way of compensating for the loss. Thus, the association found between number of pregnancies and adverse pregnancy outcomes could be as a result of “reverse causation.”

Another limitation worth considering is that the sample selection in this study is very restrictive as it only includes last pregnancies/births in the last 3 years prior to the survey (2004–2007). This is also apparent in the final study sample, which was a very young population (average 22 years, median 22 years) compared to the average age of 29 years for respondents to the entire women’s questionnaire (Ghana Statistical Service (GSS) Ghana Health Service (GHS), and Macro International 2009). We also observed from the data that many pregnancies happened at a relatively low age. This could be a marker of socio-economic status and possibly linked to the heat exposure measure. An example is being forced to work outdoors in high ambient heat to assure food security. Thus, there could be a potential risk of over-adjustment by adjusting for both age a pregnancy and number of pregnancies.

Lastly, on the suitability of making this type of analyses using available dataset, we observed that this study lacked data on some factors that are known from previous studies to impact on the susceptibility of an individual to outdoor heat exposure such as time spent inside and outside at the home, work, and recreational environments that influence daily physical activity level, and adaptation behaviors such as air conditioner use, hydration, and removal from the hot environment (Anderson and Bell 2009; Medina-Ramón and Schwartz 2007; O’Neill et al. 2005; Van Zutphen et al. 2012).

Figures 2 and 3 show the very narrow range of monthly and yearly heat levels in Ghana. Thus, the lack of statistically significant results may be due to the very similar heat exposure situations for all women. Therefore, additional analysis using data from a different country with wide ambient heat variations (e.g., very hot summers and cool winters) will be essential for further studies on this topic.

The use of monthly average WBGT makes the range of values very small, which may explain the lack of any significant effect. It may well be that individual superhot days during key periods of pregnancy is what may cause heat effects on the fetus. This is supported by evidence from a recent study in northeast Ghana, which indicated that the WBGT in the working environment of farmers could peak at 33.0 to 38.1 °C during the middle of the day and dropped to 14.0–23.7 °C in the early mornings during the same season (Frimpong et al. 2017). Generally, the yearly maximum WBGT (WBGT max) values had a very narrow variation (ranged from 26.1 to 27.5) compared to the yearly average (WBGT mean) (ranged from 24.0 to 26.1 °C) over the study period (2004–2007), making the WBGT mean a preferred index for this particular study.

We narrowed down the exposure window in the analyses by time and place, both of which are relevant for better estimation of exposure to high ambient heat (Basu 2009). However, we found a stronger effect size when we focused the exposure lens by using only the four selected regions, but rather negligible effect when we focused on both narrow time window (exposure at second month of pregnancy) and place (Table 4). This could be as a result of the difficulties in accurately linking the heat exposure values to the specific geographic location of the woman at the second month of pregnancy. Short period relocations during early or late pregnancies are possible. The selective analyses of the four administrative regions, using two regions in northern Ghana, which are further apart from the two selected southern regions could have partly reduced the bias in misclassifying heat exposure, since we expect that mass mobility between the extreme north and the extreme south regions is less likely in a short time period. Future analysis of this sort will benefit from inclusion of specific geographical data into surveys regarding maternal health and pregnancy outcomes that could be linked to remote sensing data.

Our intention with study was to empirically evaluate an attempt to use existing epidemiological information from a national survey in a low-income setting, combined with existing information from routine weather observation, which has been made available through the HOTHAPS initiative. If this is a feasible way forward, many similar studies could be made in similar countries/geographical areas, since similar data already exists, e.g., through the existence of data generated by more than a hundred Demographic Health and Surveillance Sites (DHSS), almost exclusively in low-income settings around the world. Even if our approach could be deemed as insufficient in comparison with well-designed study utilizing the specific state-of-the-art measurements, we think that it can generate a better founded discussion regarding which data would be needed, and perhaps how the analytical models could be developed, for making cost-efficient use of already existing information, i.e., we think that our study could contribute to further steps forward regarding research on the effect of ambient high temperatures on pregnancy outcome and on health outcomes in general. This is an area where more epidemiological studies are warranted, in order to estimate important aspects of the impact of global climate change on health.

## Conclusion

Environmental heat exposures may be associated with adverse pregnancy outcomes. Historical records of routine observations from existing weather stations can be used for epidemiological studies on the health effects of heat, although data from properly and purposively designed studies might be more suitable for further studies.
